# A nomogram combining inflammatory markers and clinical factors predicts survival in patients with diffuse glioma

**DOI:** 10.1097/MD.0000000000027972

**Published:** 2021-11-24

**Authors:** Ping Yan, Jian-Wen Li, Li-Gen Mo, Qian-Rong Huang

**Affiliations:** Department of Neurosurgery, Guangxi Medical University Cancer Hospital, Nanning Guangxi, P.R. China.

**Keywords:** glioma, inflammatory marker, nomogram, prognosis

## Abstract

In this study, we aimed to investigate the prognostic value of neutrophil/lymphocyte ratio (NLR), monocyte/lymphocyte ratio (MLR), and platelet/lymphocyte ratio (PLR) in diffuse glioma, and to establish a prognostic nomogram accordingly.

The hematologic and clinicopathological data of 162 patients with primary diffuse glioma who received surgical treatment from January 2012 to December 2018 were retrospectively analyzed. Receiver operator characteristic (ROC) curve was carried out to determine the optimal cut-off values for NLR, MLR, PLR, age, and Ki-67 index, respectively. Kaplan–Meier method was used to investigate the correlation between inflammatory indicators and prognosis of glioma patients. Univariate and multivariate Cox regression were performed to evaluate the independent prognostic value of each parameter in glioma. Then, a nomogram was developed to predict 1-, 3-, and 5-year postoperative survival in diffuse glioma patients based on independent prognostic factors. Subsequent time-dependent ROC curve, calibration curve, decision curve analysis (DCA), and concordance index (C-index) were performed to assess the predictive performance of the nomogram.

The Kaplan–Meier curve indicated that patients with high levels of NLR, MLR, and PLR had a poor prognosis. In addition, we found that NLR level was associated with World Health Organization (WHO) grade and IDH status of glioma. The multivariate Cox analysis indicated that resection extent, WHO grade, and NLR level were independent prognostic factors, and we established a nomogram that included these three parameters. The evaluation of the nomogram indicated that the nomogram had a good predictive performance, and the addition of NLR could improve the accuracy.

NLR, MLR, and PLR were prognostic factors of diffuse glioma. In addition, the nomogram including NLR was reliable for predicting survival of diffuse glioma patients.

## 1 Introduction

Gliomas originate in the central nervous system and are the most common neuroepithelial carcinomas, accounting for approximately 80% of primary malignant brain tumors.^[1,2]^ There has been some progress in the treatment of glioma over the past few decades, but the prognosis remains poor.^[3,4]^ Although some clinicopathological factors of glioma (such as tumor grade, age at diagnosis, tumor resection extent, IDH status, etc) are considered to be closely related to prognosis, the prognosis of glioma is still highly variable.^[5]^ Hence, glioma patients urgently need a more accurate prognostic indicator.

Recently, increasing evidence has shown that preoperative hematologic indicators are not only related to nutrition and coagulation, but also related to tumor progression, including tumor growth, proliferation, metastasis, and even recurrence.^[6–8]^ In addition, many studies have shown that inflammatory markers are prognostic factors for glioma.^[9–11]^ However, few studies have integrated inflammatory markers and clinical data into an overall understanding of glioma prognosis, making these inflammatory markers less useful in guiding clinical treatment. Thus, we believe that it is necessary to further analyze the application value of these inflammatory markers by combining clinical features. The aim of our study was to explore the prognostic roles of NLR, MLR, and PLR in diffuse glioma (WHO grade II-IV) patients, and to construct a nomogram model combining inflammatory markers with clinicopathologic features to predict 1-, 3-, and 5-year postoperative survival.

## 2 Materials and methods

### 2.1 Study population

In this study, patients with primary diffuse glioma who underwent surgery in the Department of Neurosurgery of Guangxi Medical University Cancer Hospital from January 2012 to December 2018 were retrospectively reviewed. The inclusion criteria were as follows:

(1) Diagnosis was confirmed by histopathology.
(2) Peripheral blood data were available preoperatively, including neutrophil, lymphocyte, monocyte, and platelet counts.
(3) No signs of active infection, hematologic disease, or extracranial tumor.
(4) No steroid treatment was given before peripheral blood examination. All patients included in this study signed informed consent, and the study was approved by the Ethics Committee of Guangxi Medical University Cancer Hospital.

### 2.2 Data collection and hematological examination

The clinicopathological features of the patients were collected from medical records, including age, gender, isocitrate dehydrogenase (IDH) status, WHO grade, Ki-67 index, resection extent (gross total resection or incomplete resection), and postoperative adjuvant therapy. Preoperative peripheral blood data, including neutrophil (10^9^ cells/L), monocyte (10^9^ cells/L), lymphocyte (10^9^ cells/L), and platelet (10^9^ cells/L) were also collected. NLR (neutrophil/lymphocyte ratio), MLR (monocyte/lymphocyte ratio), and PLR (platelet/lymphocyte ratio) were then calculated for each patient. The interval from the date of surgery to death or last follow-up (February 2021) was defined as overall survival (OS).

### 2.3 Statistical analysis

Statistical analysis and graphical analyses in this study were carried out using R software (version 3.6.3). Wilcox test was used to measure the differences between 2 groups. The optimal cut-off values of NLR, MLR, PLR, age, and Ki-67 index were analyzed by ROC curve. Differences of OS between the 2 groups were compared by Kaplan–Meier survival curve and log-rank test. Univariate and multivariate Cox regression were carried out to evaluate the independent prognostic value of each parameter in glioma. We then constructed a prognostic nomogram model using rms R package based on independent prognostic factors. Subsequent time-dependent ROC curve, calibration curve, DCA, and C-index were used to assess the predictive performance of the nomogram. *P* value < .05 was considered statistically significant.

## 3 Results

### 3.1 Patients characteristics

In this study, 162 patients with primary diffuse glioma were included, including 88 males (54.32%) and 74 females (45.68%). The clinicopathological features of this cohort are summarized in Table 1. The age ranged from 7 to 82 years, with a median age of 45 years. According to the ROC curve, the optimal cut-off for age was 47 years, and there were 90 (55.56%) patients with age ≤ 47 years. In this cohort, 71 (43.83%) cases were WHO grade II, 33 (20.37%) cases were grade III, and 58 (35.80%) cases were grade IV. The cut-off value for Ki-67 index was 10%, and there were 59 (36.42%) patients with Ki-67 index ≤ 10%. The number of patients with IDH mutation, wildtype, and undefined was 46 (28.40%), 52 (32.10%), and 64 (39.50%), respectively. According to postoperative imaging examination, gross total resection was performed in 72 patients (44.44%). For postoperative adjuvant therapy, 59 (36.42%) patients received radiotherapy and 72 (44.44%) patients received chemotherapy.

**Table 1:** Clinical characteristics of 162 patients in this cohort.

Characteristics	Median (range)	Number (%)
Gender		
Male		88 (54.32)
Female		74 (45.68)
Age, yr	45 (7–82)	
≤ 47		90 (55.56)
> 47		72 (44.44)
WHO Grade		
II		71 (43.83)
III		33 (20.37)
IV		58 (35.80)
Ki-67 index	20% (1–85%)	
≤ 10%		59 (36.42)
> 10%		103 (63.58)
IDH status		
Mutant		46 (28.40)
Wildtype		52 (32.10)
Undefined		64 (39.50)
Resection extent		
Gross total		72 (44.44)
Incomplete		90 (55.56)
Radiotherapy		
Yes		59 (36.42)
No		103 (63.58)
Chemotherapy		
Yes		72 (44.44)
No		90 (55.56)
NLR	2.44 (0.73–22.44)	
≤ 2.78		98 (60.49)
> 2.78		64 (39.51)
MLR	0.240 (0.042–1.922)	
≤ 0.235		75 (46.30)
> 0.235		87 (53.70)
PLR	134.6 (67.2–653.2)	
≤ 134.4		77 (47.53)
> 134.4		85 (52.47)

**Figure 1:**
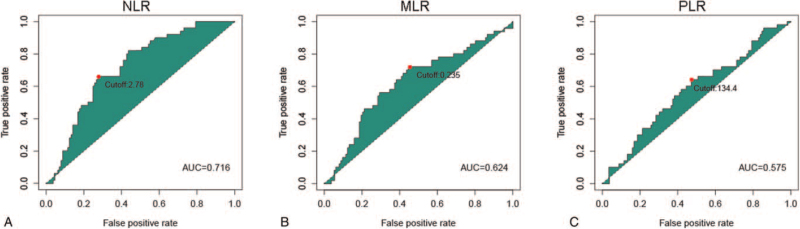
ROC curves analysis for optimal cut-off values for (A) NLR, (B) MLR, and (C) PLR. The optimal cut-off values for NLR, MLR and PLR were 2.78, 0.235, and 134.4, respectively.

According to the ROC curves for survival prediction, the optimal cut-off values of NLR (AUC: 0.716), MLR (AUC: 0.624), PLR (AUC: 0.575), were determined to be 2.78, 0.235, and 134.4, respectively (Fig. 1). We then divided patients into high level and low level according to the cut-off values of these 3 inflammatory markers, respectively.

### 3.2 Relationship of NLR, MLR, and PLR with glioma features

**Figure 2:**
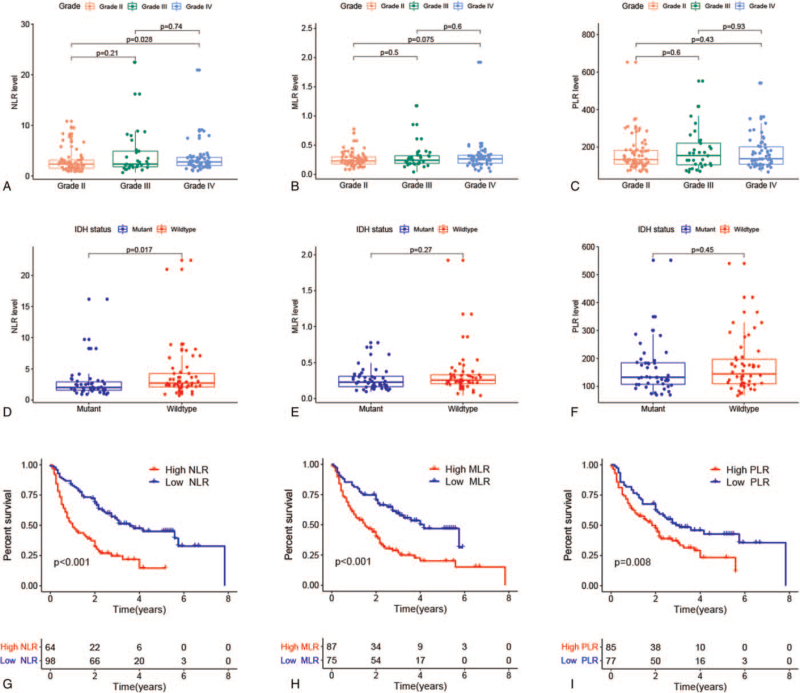
Relationship of NLR, MLR, and PLR with clinicopathologic features and prognosis of glioma. Correlation of NLR, MLR, and PLR with (A--C) WHO grade and (D--F) IDH status. (G--I) Kaplan--Meier curves were used to compare the OS of patients with high and low levels of NLR, MLR ,and PLR, respectively.

We analyzed the distribution of NLR, MLR, and PLR according to the WHO grade and IDH status. The results showed that the NLR levels in grade IV were significantly higher than those in grade II (*P* = .028, Fig. 2A). In addition, patients with IDH wildtype had significantly increased NLR levels compared with patients with IDH mutation (*P* = .017, Fig. 2D). These results suggested that the level of NLR might be associated with the malignancy of glioma. However, no significant differences were observed in the results of MLR (Fig. 2B, E) and PLR analyses (Fig. 2C, F).

### 3.3 Prognostic value of NLR, PLR, and MLR in glioma

We next explored the correlation between these inflammatory markers and OS in glioma patients. The Kaplan–Meier curve indicated that patients with high NLR (*P* < .001, Fig. 2G) had a poor prognosis. Similar results were observed in PLR and MLR. Higher levels of MLR (*P* < .001, Fig. 2H) and PLR (*P* = .008, Fig. 2I) were associated with worse clinical outcomes. Furthermore, univariate and multivariate Cox regression analyses were performed to assess whether these inflammatory markers could be independent predictors of glioma prognosis. In univariate analysis, gender, age, WHO grade, Ki-67 index, IDH status, resection extent, NLR, MLR, and PLR were strongly associated with OS of glioma (*P* < .05). According to multivariate analysis, gross total resection [hazard ratio (HR) = 0.465, 95% confidence interval (95% CI) = 0.300–0.721, *P* < .001], WHO grade III (HR = 1.935, 95% CI = 1.024–3.655, *P* =  .042), grade IV (HR = 2.425, 95% CI = 1.192–4.936, *P* = .015), and NLR > 2.78 (HR = 2.637, 95% CI = 1.478–4.707, *P* = .001) were independent prognostic factors in patients with glioma (Table 2).

**Table 2:** Results of the univariate and multivariate Cox analysis of the OS in glioma patients.

	Univariate analysis		Multivariate analysis	
Covariates	HR (95% CI)	*P*	HR (95% CI)	*P*
Gender				
Female vs Male	1.693 (1.122–2.554)	.012	1.123 (0.705–1.787)	.626
Age, years				
≤ 47 vs > 47	2.558 (1.699–3.852)	< .001	1.418 (0.860–2.336)	.171
Resection extent				
Incompletion vs Gross total	0.546 (0.360–0.829)	.005	0.465 (0.300–0.721)	< .001
WHO grade				
II vs III	2.209 (1.252–3.899)	.006	1.935 (1.024–3.655)	.042
II vs IV	3.943 (2.462–6.316)	< .001	2.425 (1.192–4.936)	.015
Ki-67 index				
≤ 10% vs > 10%	2.266 (1.451–3.540)	< .001	1.584 (0.865–2.900)	.136
IDH status				
Mutant vs Undefined	1.350 (0.798–2.282)	.263	0.842 (0.456–1.558)	.585
Mutant vs Wildtype	2.148 (1.266–3.647)	.005	0.782 (0.387–1.579)	.493
Radiotherapy				
No vs Yes	0.797 (0.526–1.207)	.284		
Chemotherapy				
No vs Yes	0.881 (0.593–1.309)	.530		
NLR				
≤ 2.78 vs > 2.78	2.643 (1.767–3.954)	< .001	2.637 (1.478–4.707)	.001
MLR				
≤ 0.235 vs > 0.235	2.335 (1.540–3.542)	< .001	1.626 (0.964–2.743)	.069
PLR				
≤ 134.4 vs > 134.4	1.710 (1.141–2.563)	.009	0.695 (0.399–1.212)	.199

CI = confidence interval, HR = hazard ratio.

### 3.4 Construction and evaluation of a nomogram model

**Figure 3:**
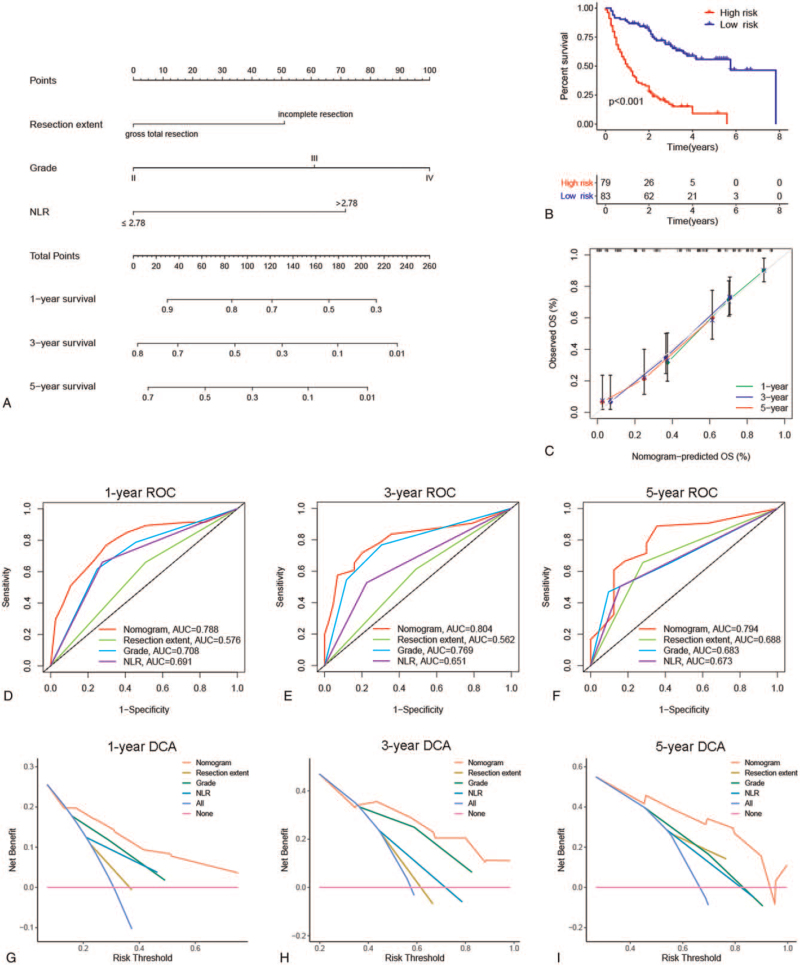
Construction and evaluation of the prognostic nomogram model. (A) Nomogram was established to predict OS of glioma patients. (B) The Kaplan--Meier curve shows difference in OS between the high-risk and low-risk groups. (C) Calibration curves were used to compare nomogram prediction and actual observation. (D--F) Time-dependent ROC curves were used to assess the accuracy of predicting 1-, 3-, and 5-year survival. (G--I) Decision curve analysis for evaluating the net clinical benefit.

To further investigate the clinical application of these inflammatory markers, we constructed a nomogram model for glioma based on the independent prognostic factors (resection extent, WHO grade, and NLR) to predict the 1-, 3-, and 5-year postoperative survival of glioma patients (Fig. 3A). On the basis of the median risk score calculated by the nomogram, we divided this cohort into 2 subgroups (low-risk and high-risk). The survival curve showed significant differences in OS between the 2 groups, suggesting that this nomogram can help clinicians accurately identify glioma patients with poor clinical outcome (*P* < .001, Fig. 3B). The calibration curve indicated that the nomogram model prediction was very close to the actual observation (Fig. 3C). The AUCs of the nomogram for predicting 1-, 3-, and 5-year survival were 0.788, 0.804, and 0.794, respectively, which were higher than other traditional clinical parameters (Fig. 3D--F). Furthermore, DCAs were carried out to evaluate the net clinical benefit, and we found that the nomogram model provided more benefit than single independent prognostic factors in predicting OS (Fig. 3G--I). We also calculated the C-index of the nomogram and clinical indicators. The C-index of the nomogram was 0.724, and the C-index of the nomogram was 0.674 when only WHO grade and resection degree were involved, indicating that the nomogram had a better predictive effect after including NLR.

## 4 Discussion

In general, surgical resection and postoperative adjuvant chemotherapy and radiotherapy are conducive to a better prognosis for malignant glioma patients. However, outcomes varied widely among patients receiving the same treatment. Hence, in order to accurately assess the prognosis of glioma in clinic, a more accurate prediction indicator is urgent. It is worth noting that many studies have shown that inflammatory factors are related to the development and prognosis of malignant glioma.^[12–14]^ Neutrophils, in particular, are often closely associated with poor prognosis in glioma patients.^[15–17]^

Inflammation is related to the tumor microenvironment.^[18]^ The tumor microenvironment refers to the environment surrounding the tumor, which is mainly composed by immune cells, endothelial cells, and inflammatory mediators. It is characterized by continuous inflammation, which has properties of inflammatory and immunosuppressive, and is regarded as an “unhealable wound.”^[19–21]^ In the tumor microenvironment, inflammation can consume lymphocytes and reduce the body's immune response to malignant tumor cells, then tumors occur.^[22]^ At the same time, tumor cells release a large number of chemokines making immune cells migrate into them to promote the production of inflammatory mediators and regulate tumor progression.^[20]^ The relatively elevated neutrophils can enhance the production of inflammatory mediators, and even cause the instability and mutation of the body's genetic status bring the function of DNA repair decrease.^[8,23,24]^ In addition, eosinophils, macrophages, and platelets are all related to tumor progression.^[25–29]^

High NLR level has been identified as an independent risk factor for OS in a variety of malignancies, including colorectal cancer,^[30]^ ovarian cancer,^[31,32]^ breast cancer,^[33]^ and pancreatic cancer.^[34,35]^ The interaction between inflammation and cancer shows that inflammation may drive the secretion of growth factors and pro-angiogenesis factors, induce tumor cell invasion and metastasis, and lead to tumor progression.^[36,37]^ Lymphocytes play an important role in the anti-tumor immunity process, which can inhibit tumor proliferation and metastasis.^[18]^ Therefore, NLR is often used as an indicator for the balance between inflammation and immune response.^[6]^ In this study, we found that the level of NLR was correlated with WHO grade and IDH status of glioma. Moreover, our data also showed that glioma patients with NLR > 2.78 had a significantly shorter OS, and NLR > 2.78 was an independent risk factor for glioma prognosis, which was consistent with previous findings.

Some studies have found that elevated PLR, MLR are indicators of poor prognosis in patients with cancer.^[38–41]^ Platelets promote angiogenesis, adhesion, and invasion through the secretion of vascular endothelial growth factor (VEGF) and platelet-derived growth factor (PDGF). Platelets not only promote tumor growth, but also promote the infiltration of other immune cells, such as neutrophils and lymphocytes, into tumor tissue and trigger further inflammatory progress.^[41–43]^ Monocytes can differentiate into macrophages, and macrophages can also promote the extravasation, survival, and metastasis of cancer cells.^[44,45]^ Lymphocytes are the main component of immune defense against malignant tumor, which can induce cell death and inhibit tumor cell proliferation and migration.^[40,46]^ Therefore, the level of lymphocytes can reflect the immune status to some extent. In the present study, there was a significant difference in OS time between patients with high and low group of PLR and MLR. However, the multivariate analysis showed that PLR and MLR were not independent prognostic factors, which may be related to the interaction between neutrophils, lymphocytes, and monocytes.^[40,47,48]^

In recent years, more and more evidence has shown that nomogram is better than traditional methods in predicting cancer patient prognosis.^[49]^ Previous studies have constructed a nomogram model for glioma prognosis by combining inflammatory indicators and clinical features, and have shown good predictive performance, but the evaluation methods of the model in these studies are limited.^[17,50]^ In our study, we constructed a nomogram to predict OS for diffuse glioma based on independent prognostic factors (resection extent, WHO grade, and NLR), and more importantly, we assessed the predictive power of the nomogram using more comprehensive methods, including survival analysis, calibration curve, ROC curve, C-index, and DCA. In addition, the parameters contained in our nomogram are easy to obtain in the clinic, and have the characteristics of low cost and convenience, which can provide reference for the individualized treatment of patients with diffuse glioma.

Several limitations of this study should be considered. First of all, as a single-center retrospective study, this study is inevitably subject to selection bias. Second, the detection rate of IDH status was low in this cohort. In addition, the lack of other important glioma molecular markers, such as MGMT promoter methylation status and 1p19q co-deletion status, may also affect the construction of the nomogram. Finally, our predicted OS is consistent with the actual observed OS, but the accuracy of nomogram needs further external validation.

In conclusion, high level of NLR, MLR, and PLR was associated with poor prognosis in patients with diffuse glioma, and NLR level was an independent prognostic factor. In addition, the nomogram including NLR was reliable for predicting survival of diffuse glioma patients.

## Author contributions

**Conceptualization:** Li-Gen Mo, Qian-Rong Huang.

**Data curation**: Ping Yan, Jian-Wen Li.

**Formal analysis**: Jian-Wen Li, Qian-Rong Huang.

**Investigation**: Ping Yan, Qian-Rong Huang.

**Methodology**: Ping Yan, Qian-Rong Huang.

**Project administration:** Li-Gen Mo, Qian-Rong Huang.

**Resources:** Li-Gen Mo, Qian-Rong Huang.

**Software**: Ping Yan, Jian-Wen Li.

**Validation**: Ping Yan, Qian-Rong Huang.

**Visualization**: Jian-Wen Li, Qian-Rong Huang.

**Writing – original draft**: Ping Yan, Qian-Rong Huang.

**Writing – review & editing:** Li-Gen Mo, Qian-Rong Huang.
